# Thermal stable properties of solid hybrid nanoparticles for mixed convection flow with slip features

**DOI:** 10.1038/s41598-022-20974-2

**Published:** 2022-09-30

**Authors:** Liaquat Ali Lund, Maha M. A. Lashin, Ubaidullah Yashkun, Kamel Guedri, Sami Ullah Khan, M. Ijaz Khan, Omar T. Bafakeeh, Poom Kumam

**Affiliations:** 1 School of Quantitative Sciences, Universiti Utara Malaysia, 06010 Sintok, Kedah Malaysia; 2 College of Engineering, Princess Nourah bint Abdulrahman University, Riyadh, Saudi Arabia; 3 Department of Mathematics and Social Sciences, Sukkur IBA University, Sukkur, Pakistan; 4 Mechanical Engineering Department, College of Engineering and Islamic Architecture, Umm Al-Qura University, P.O. Box 5555, Mecca, 21955 Saudi Arabia; 5 Department of Mathematics, COMSATS University Islamabad, Sahiwal, 57000 Pakistan; 6 Department of Mathematics and Statistics, Riphah International University I-14, Islamabad, 44000 Pakistan; 7 Mathematical Modelling and Applied Computation Research Group (MMAC), Department of Mathematics, King Abdulaziz University, Jeddah, Saudi Arabia; 8 Department of Industrial Engineering, Jazan University, Jazan, 82822 Saudi Arabia; 9 Center of Excellence in Theoretical and Computational Science (TaCS-CoE) and KMUTT Fixed Point Research Laboratory, Room SCL 802 Fixed Point Laboratory, Science Laboratory Building, Departments of Mathematics, Faculty of Science, King Mongkut’s University of Technology Thonburi (KMUTT), 126 Pracha-Uthit Road, Bang Mod, Thung Khru, Bangkok, 10140 Thailand; 10 Department of Medical Research, China Medical University Hospital, China Medical University, Taichung, 40402 Taiwan

**Keywords:** Engineering, Mathematics and computing

## Abstract

Following to improved thermal impact of hybrid nanomaterials, wide range applications of such materials is observed in the thermal engineering, extrusion systems, solar energy, power generation, heat transfer devices etc. The hybrid nanofluid is a modified form of nanofluid which is beneficial for improving energy transfer efficiency. In current analysis, the solid nanoparticles aluminium ($\phi_{{{\text{Al}}_{2} {\text{O}}_{3} }}$) and copper ($\phi_{{{\text{Cu}}}}$) have been mixed with water to produce a new hybrid nanofluid. The investigation of a steady two-dimensional mixed convection boundary layer flow of the resultant hybrid nanofluid on a vertical exponential shrunk surface in the existence of porous, magnetic, thermal radiation, velocity, and thermal slip conditions is carried out. Exponential similarity variables are adopted to transform the nonlinear partial differential equation into a system of ordinary differential equations which has been then solved by employing the shooting method in Maple software. The obtained numerical results such as coefficient of skin friction $f^{\prime \prime } \left( 0 \right)$, heat transfer rate $- \theta^{\prime } \left( 0 \right)$, velocity $f^{\prime } \left( \eta \right)$ and temperature $\left( {\theta \left( \eta \right)} \right)$ distributions are presented in the form of different graphs. The results revealed that duality exists in solution when the suction parameter $S \ge S_{ci}$ in assisting flow case. Due to non-uniqueness of solutions, a temporal stability analysis needs to be performed and the result indicates that the upper branch is stable and realizable compared to the lower branch.

## Introduction

From last few years, the investigation of fluid dynamics has attracted considerable much attention amongst analysts and scholars from various fields because of its enormous potential applications in engineering, designing, science, innovation, and technology. The most frequently discussed topics are related to boundary layer flow which was initially discovered by Ludwig Prandtl. Since then, many scholars had attempted to study numerous types of Newtonian and non-Newtonian fluids with different characteristics and sheets. Hakeem et al.^1^ investigated the effect on heat transfer of a Casson fluid by using of inclined magnetized field. They concluded that the aligned angle role in regulating the magnetic strength in the Casson fluid flow area is essential. When the aligned magnetic field values increase, both Nusselt number and the coefficient of skin friction decrease, while the temperature of the non-dimensional surface raises. Mandal et al.^2^ examined the thermal radiation effect on the micropolar fluid. They deduced that as the value of mixed convection parameter inclines, the flow velocity tends to increase, but both temperature and angular velocity seem to decline. Meanwhile, Shahzad et al.^3^ premeditated the effect of heat transfer of axisymmetric flow of magnetized fluid on an exponentially stretching surface. It is noticed that the velocity of radial increases when the suction and magnetic parameters are increased.

In convection, the heat transfer occurs through mass movement of fluid molecules (gases or liquids). It takes place above the hot surface where the heated fluid molecules become less dense and move from one place to another, taking heat within them due to the difference in temperature. convection is a major source of heat transfer that happens through diffusion or advection or both^4^. For an example, the convection exists in the cooling of the electronic parts of the computer. A small fan is installed to the side or rear of chassis to cool the electronic components with openings on the side surface for easy air circulation. There are three kinds of convection. In free convection, the flow of heat transfer takes place due to body forces that happen because of density changes that arise due to the temperature difference in the flow filed. This is an important mode of heat transfer and widely used in engineering and industrial applications. Forced convection, on the hand, occurs when the temperature of the solid surface and the fluid are different. The heat is transmitted as a forced convection from the hotter to the colder regime. According to Waini et al.^5^, “the fluid motion for the case of forced convection is due to an external motive source such as a fan or pump. The phenomena of forced convection are also very important and have many applications in industry such as the radiator system in vehicles, heating, and cooling of parts of the body by blood circulation”. Meanwhile, mixed convection is a mixture of forced and free convection. It is a very efficient mechanism of heat transmission that occurs in a wide variety of transport processes in both engineered devices and nature. In mixed convection both free and forced convection act together in the process of heat transfer^6^. It is most likely that Merkin^7^ is the first researcher who considered a mixed convection effect on boundary layer flow for multiple solutions. Later in 1986, he extended his work to porous medium and found dual solutions^8^. Ahmad et al.^9^ further explored the concept of mixed convection flow on nanofluid and stated that there is no uniqueness of solutions within a limited range of parameters.

Recently, invention of the advanced heat transfer fluids acquired attention in the field of science and technology. One of these fluids is hybrid nanofluid which is also referred as a modern type of nanofluid. Since nanofluid is a blend of solid nanoparticles in the base fluid as stated in Choi and Eastman^10^ while hybrid nanofluid is the blend of nanoparticles in a base nanofluid in which nanofluid particles should be distinct. Avramenko and Shevchuk^11^ discussed the self-similar approach to the heat and mass transfer phenomenon associated to the nanofluid problem. Avramenko and Shevchuk^12^ reported the thermal impact of nanomaterials in absence of condensation and boiling phenomenon. Gowda et al.^13^ observed the vertically moving disk supported with decomposition of hybrid nanoparticles. Kumar et al.^14^ studied the cylindrical flow of ferromagnetic nanoparticles with significant contribution of magnetic dipole. Radhika et al.^15^ discussed the hybrid nanoparticles suspension with dust particles confined by melting surface. Kumar et al.^16^ discussed the rotating surface flow in upward and downward moving disk subject to the hybrid nanofluids. Gowda et al.^17^ contributed the Dufour features towards the nanofluid numerically. The Marangoni convection flow of nanofluid with binary chemical reaction was evaluated by Gowda et al.^18^. Haq et al.^19^ depicted a theoretical thermal visualization of hybrid nanofluid problem with permeable cylinder. The fractional based mathematical model for hybrid nanofluid has been worked out by Wang et al.^20^. Lund et al.^21^ performed a stability measurement of nanofluid via shrinking surface. Yan et al.^22^ proposed the heating impact of nanofluid with imposition of multiple slip effects.

The aim of current continuation is to express the thermal dynamic of hybrid nanofluid due to vertically space when multiple slip effects are significant. The novelty of current work is justified as:

- The thermal impact of hybrid nanofluid with utilization of copper and aluminium oxide nanoparticles with water base fluid.
- The mixed convection, thermal raidation and magnetic force influences are contributed.
- The hybrid nanofluid is impacted with multiple velocity and thermal flow constraints. The motivations for consideration such slip features are associated to control of hybrid nanofluid velocity and thermal performances.
- The stability of hybrid nanoparticles is evaluated and evaluated. It is emphasized that various thermal models on nanofluid are available in the literature, however, the stability framework of such models is not ensured in most of investigations.
- The velocity and thermal profiles are observed in distinct flow regimes.
- The numerical prediction of flow model are captured with shooting technique.

## Mathematical description of problem

**Figure 1:**
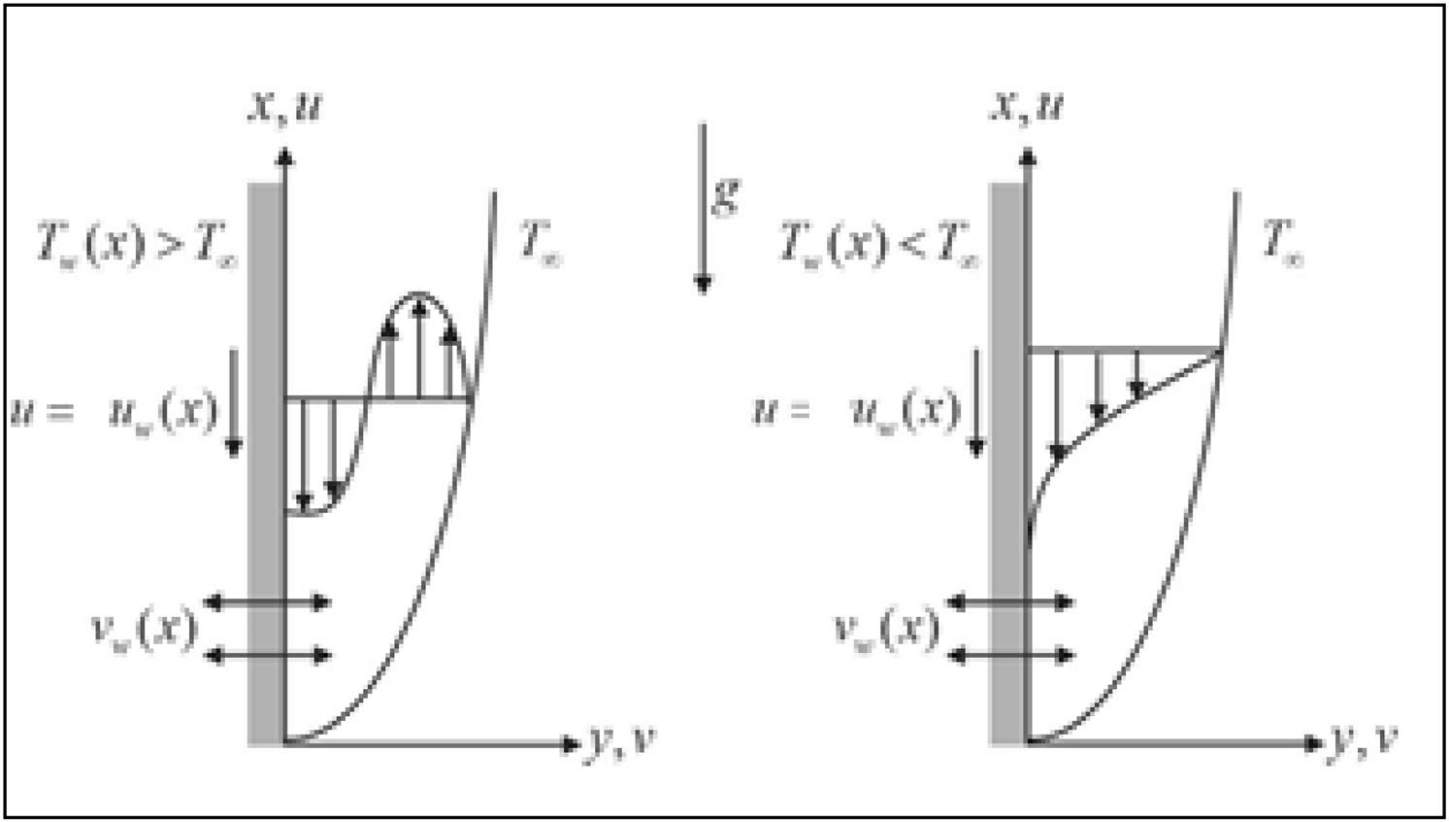
Physical model of the problem.

Let us consider 2D, steady mixed convection and incompressible flow of hybrid nanofluid with effect of porous medium, thermal radiation over a vertical exponentially shrinking sheet (refer to Fig. 1). The governing equations are simplified via boundary layer theory^23^. Moreover, the uniform magnetic field of strength $B$ is applied normal to a shrinking sheet. The governance model with all assumptions are as follows^20–22^:

1 $$\frac{\partial u}{{\partial x}} + \frac{\partial v}{{\partial y}} = 0$$

2 $$u\frac{\partial u}{{\partial x}} + v\frac{\partial u}{{\partial y}} = \frac{{\mu_{hnf} }}{{\rho_{hnf} }}\frac{{\partial^{2} u}}{{\partial y^{2} }} + \beta_{hnf} g\left( {T - T_{\infty } } \right) - \frac{1}{{\rho_{hnf} }}\left( {\frac{{\mu_{hnf} }}{{K^{*} }} + \sigma_{hnf} B^{2} } \right)u$$

3 $$u\frac{\partial T}{{\partial x}} + v\frac{\partial T}{{\partial y}} = \left[ {\frac{{k_{hnf} }}{{\left( {\rho c_{p} } \right)_{hnf} }} + \frac{{16\sigma_{1} T_{\infty }^{3} }}{{3k^{*} \left( {\rho c_{p} } \right)_{hnf} }}} \right]\frac{{\partial^{2} T}}{{\partial y^{2} }}$$

with boundary conditions^20,21^:

4 $$\left\{ {\begin{array}{*{20}l} {v = v_{w} \left( x \right), u = u_{w} + A\vartheta_{f} \frac{\partial u}{{\partial y}}, T = T_{w} + D\frac{\partial T}{{\partial y}}} \hfill & {{\text{as}}\;y = 0} \hfill \\ {u \to 0, T \to T_{\infty } ,} \hfill & {{\text{as}}\;y \to \infty } \hfill \\ \end{array} } \right..$$

The velocities of $y\,\,{\text{and}}\,x$ axis are denoted by $v$ and $u$ accordingly, $K^{*} = K_{0}^{*} e^{ - x/l}$ shows the permeability of porous medium, $B = B_{0} e^{x/2l}$ is the magnetic field along with constant magnetic strength $e^{x/2l}$, $T$ is the temperature of fluid, $T_{w} \left( x \right) = T_{\infty } + T_{0} e^{2x/l}$ is the temperature of surface where $T_{\infty }$ is free stream temperature, $\left( {\rho c_{p} } \right)_{hnf} ,\rho_{hnf} ,{ }\sigma_{hnf} , k_{hnf} ,$ and $\mu_{hnf} ,$ are the effective heat capacity, density, electrical conductivity, thermal conductivity and viscosity of the considered hybrid nanofluid. In addition, $u_{w} = - U_{w} e^{x/l}$ is the surface velocity,$A = A_{1} e^{ - x/2l}$ is velocity slip factor, $D = D_{1} e^{ - x/2l}$ is thermal slip factor, and $v_{w} = \sqrt {\frac{{\vartheta_{f} U_{w} }}{2l}} e^{x/2l} S$ where $S$ is the parameter of blowing/suction.

In this study the thermophysical properties of nanomaterials, base fluid, and hybrid nanofluid are used. In relation to, Tables 1 and 2 are presented^20,21^.

**Table 1:** Thermophysical features of hybrid nanofluid^20,21^.

Properties	Hybrid nanofluid
Dynamic viscosity	$\mu_{hnf} = \frac{{\mu_{f} }}{{\left( {1 - \phi_{{{\text{Al}}_{2} {\text{O}}_{3} }} } \right)^{2.5} \left( {1 - \phi_{{{\text{Cu}}}} } \right)^{2.5} }}$
Density	$\rho_{hnf} = \left( {1 - \phi_{{{\text{Cu}}}} } \right)\left[ {\left( {1 - \phi_{{{\text{Al}}_{2} {\text{O}}_{3} }} } \right)\rho_{f} + \phi_{{{\text{Al}}_{2} {\text{O}}_{3} }} \rho_{{{\text{Al}}_{2} {\text{O}}_{3} }} } \right] + \phi_{{{\text{Cu}}}} \rho_{{{\text{Cu}}}}$
Thermal conductivity	$k_{hnf} = \frac{{k_{{{\text{Cu}}}} + 2k_{nf} - 2\phi_{{{\text{Cu}}}} \left( {k_{nf} - k_{{{\text{Cu}}}} } \right)}}{{k_{{{\text{Cu}}}} + 2k_{nf} + \phi_{{{\text{Cu}}}} \left( {k_{nf} - k_{{{\text{Cu}}}} } \right)}} \times \left( {k_{nf} } \right)$ where $k_{nf} = \frac{{k_{{{\text{Al}}_{2} {\text{O}}_{3} }} + 2k_{f} - 2\phi_{{{\text{Al}}_{2} {\text{O}}_{3} }} \left( {k_{f} - k_{{{\text{Al}}_{2} {\text{O}}_{3} }} } \right)}}{{k_{{{\text{Al}}_{2} {\text{O}}_{3} }} + 2k_{f} + \phi_{{{\text{Al}}_{2} {\text{O}}_{3} }} \left( {k_{f} - k_{{{\text{Al}}_{2} {\text{O}}_{3} }} } \right)}} \times \left( {k_{f} } \right)$
Heat capacity	$\left( {\rho c_{p} } \right)_{hnf} = \left( {1 - \phi_{{{\text{Cu}}}} } \right)\left[ {\left( {1 - \phi_{{{\text{Al}}_{2} {\text{O}}_{3} }} } \right)\left( {\rho c_{p} } \right)_{f} + \phi_{{{\text{Al}}_{2} {\text{O}}_{3} }} \left( {\rho c_{p} } \right)_{{{\text{Al}}_{2} {\text{O}}_{3} }} } \right] + \phi_{{{\text{Cu}}}} \left( {\rho c_{p} } \right)_{{{\text{Cu}}}}$
Electrical conductivity	$\sigma_{hnf} = \frac{{\sigma_{2} + 2\sigma_{nf} - 2\phi_{2} \left( {\sigma_{nf} - \sigma_{2} } \right)}}{{\sigma_{2} + 2\sigma_{nf} + \phi_{2} \left( {\sigma_{nf} - \sigma_{2} } \right)}} \times \left( {\sigma_{nf} } \right)$@ where $\sigma_{nf} = \frac{{\sigma_{1} + 2\sigma_{f} - 2\phi_{1} \left( {\sigma_{f} - \sigma_{1} } \right)}}{{\sigma_{1} + 2\sigma_{f} + \phi_{1} \left( {\sigma_{f} - \sigma_{1} } \right)}} \times \left( {\sigma_{f} } \right)$
Thermal expansion coefficient	$\left( \beta \right)_{hnf} = \left( {1 - \phi_{{{\text{Cu}}}} } \right)\left[ {\left( {1 - \phi_{{{\text{Al}}_{2} {\text{O}}_{3} }} } \right)\left( \beta \right)_{f} + \phi_{{{\text{Al}}_{2} {\text{O}}_{3} }} \left( \beta \right)_{{{\text{Al}}_{2} {\text{O}}_{3} }} } \right] + \phi_{{{\text{Cu}}}} \left( \beta \right)_{{{\text{Cu}}}}$

**Table 2:** The properties of thermos physical^20,21^.

Fluids	Copper (Cu)	Alumina (${\text{Al}}_{2} {\text{O}}_{3}$)	Water (${\text{H}}_{2} {\text{O}}$)	$\beta \times 10^{ - 5} \left( {1/{\text{K}}} \right)$
$\rho$(kg/m^3^)	8933	3970	997.1	0.85
$c_{p}$(J/kg K)	385	765	4179	1.67
*k* (W/m K)	400	40	0.613	21

The following similarity transformation variables will be adopted to convert the system of equations into ODEs^20,21^.

5 $$\psi = \sqrt {2\vartheta_{f} lU_{w} } e^{x/2l} f\left( \eta \right); \theta \left( \eta \right) = \frac{{T - T_{\infty } }}{{T_{w} - T_{\infty } }}; \eta = y\sqrt {\frac{{U_{w} }}{{2\vartheta_{f} l}}} e^{x/2l}$$

where $\psi$ represents the stream function while the velocities are as $u = \frac{\partial \psi }{{\partial y}}$ and $v = - \frac{\partial \psi }{{\partial x}}$. Put Eq. (5) in the Eqs. (2–3) yields

6 $$f^{\prime \prime \prime } - Kf^{\prime } + \xi_{1} \xi_{2} \left\{ {f^{\prime \prime } f - 2\left( {f^{\prime } } \right)^{2} + 2\lambda_{1} \left( {\beta_{hnf} /\beta_{f} } \right)\theta } \right\} - \frac{{\sigma_{hnf} }}{{\sigma_{f} }}\xi_{2} Mf^{\prime } = 0$$

7 $$\frac{1}{{Pr\xi_{3} }}\left[ {\left( {k_{hnf} /k_{f} } \right) + \frac{4Rd}{3}} \right]\theta^{\prime \prime } + \theta^{\prime } f - 4\theta f^{\prime } = 0$$

8 $$\left\{ {\begin{array}{*{20}l} {\xi_{1} = \left\{ {\left( {1 - \phi_{{{\text{Cu}}}} } \right)\left[ {1 - \phi_{{{\text{Al}}_{2} {\text{O}}_{3} }} + \phi_{{{\text{Al}}_{2} {\text{O}}_{3} }} \left( {\rho_{{{\text{Al}}_{2} {\text{O}}_{3} }} /\rho_{f} } \right)} \right] + \phi_{Cu} \left( {\rho_{Cu} /\rho_{f} } \right)} \right\}} \hfill \\ {\xi_{2} = \left( {1 - \phi_{{{\text{Cu}}}} } \right)^{2.5} \left( {1 - \phi_{{{\text{Al}}_{2} {\text{O}}_{3} }} } \right)^{2.5} } \hfill \\ {\xi_{3} = \left\{ {\left( {1 - \phi_{{{\text{Cu}}}} } \right)\left[ {1 - \phi_{{{\text{Al}}_{2} {\text{O}}_{3} }} + \phi_{{{\text{Al}}_{2} {\text{O}}_{3} }} \frac{{\left( {\rho c_{p} } \right)_{{{\text{Al}}_{2} {\text{O}}_{3} }} }}{{\left( {\rho c_{p} } \right)_{f} }}} \right] + \phi_{{{\text{Cu}}}} \frac{{\left( {\rho c_{p} } \right)_{{{\text{Cu}}}} }}{{\left( {\rho c_{p} } \right)_{f} }}} \right\}} \hfill \\ \end{array} } \right.$$

along with boundary conditions

9 $$\left\{ {\begin{array}{*{20}l} {f\left( 0 \right) = S,f^{\prime } \left( 0 \right) = - 1 + \delta f^{\prime \prime } \left( 0 \right), \theta \left( 0 \right) = 1 + \delta_{T} \theta^{\prime } \left( 0 \right)} \hfill \\ {f^{\prime } \left( \eta \right) \to 0; \theta \left( \eta \right) \to 0\;as\;\eta \to \infty } \hfill \\ \end{array} } \right.$$

where $\lambda_{1} = \frac{{g\beta_{f} T_{0} l}}{{U_{w}^{2} }}$ is the mixed parameter, $M = \frac{{2l\sigma_{f} \left( {B_{0} } \right)^{2} }}{{U_{w} \rho_{f} }}$ is a Hartmann number, $K = \frac{{l\mu_{f} }}{{U_{w} \rho_{f} K_{0}^{*} }}$ is the parameter of porosity, $\delta = A_{1} \sqrt {\frac{{\vartheta_{f} U_{w} }}{2l}}$ is the velocity slip parameter,$\delta_{T} = D_{1} \sqrt {\frac{{U_{w} }}{{2\vartheta_{f} l}}}$ is the thermal slip parameter, $Pr = \frac{{\vartheta_{f} }}{{\alpha_{f} }}$ is Prandtl number, and $Rd = \frac{{4\sigma_{1} T_{\infty }^{3} }}{{k^{*} k_{f} }}$ is thermal radiation parameter.

The substantial physical factors skin friction coefficient $C_{f}$ and local Nusselt number $Nu_{x}$ are

10 $$C_{f} = \frac{{\mu_{hnf} }}{{\rho_{f} u_{w}^{2} }}\left( {\frac{\partial u}{{\partial y}}} \right)\left| {y = 0} \right.,\quad Nu_{x} = - \frac{{xk_{hnf} }}{{k_{f} \left( {T_{w} - T_{\infty } } \right)}}\left( {\frac{\partial T}{{\partial y}}} \right)\left| {y = 0} \right.$$

By using Eq. (5) in Eq. (10) leads to

11 $$\sqrt {Re} C_{f} = \frac{1}{{\xi_{2} }} f^{\prime \prime } \left( 0 \right); \sqrt{\frac{1}{Re}} Nu_{x} = - \left[ {\frac{{k_{hnf} }}{{k_{f} }} + \frac{4Rd}{3}} \right] + \theta^{\prime } \left( 0 \right).$$

## Stability analysis

To determine the stable solution a stability analysis is executed. Temporal stability of the solutions is possible when the unsteady model of the Eqs. (2–3) are considered by introducing $\tau = \frac{{U_{w} }}{2l}e^{x/l} \cdot t$ as proposed by Weidman et al.^20^. As a result, we have

12 $$\frac{\partial u}{{\partial t}} + u\frac{\partial u}{{\partial x}} + v\frac{\partial u}{{\partial y}} = \frac{{\mu_{hnf} }}{{\rho_{hnf} }}\frac{{\partial^{2} u}}{{\partial y^{2} }} + \beta_{hnf} g\left( {T - T_{\infty } } \right) - \frac{1}{{\rho_{hnf} }}\left( {\frac{{\mu_{hnf} }}{{K^{*} }} + \sigma_{hnf} B^{2} } \right)u$$

13 $$\frac{\partial T}{{\partial t}} + u\frac{\partial T}{{\partial x}} + v\frac{\partial T}{{\partial y}} = \left[ {\frac{{k_{hnf} }}{{\left( {\rho c_{p} } \right)_{hnf} }} + \frac{{16\sigma_{1} T_{\infty }^{3} }}{{3k^{*} \left( {\rho c_{p} } \right)_{hnf} }}} \right]\frac{{\partial^{2} T}}{{\partial y^{2} }}.$$

Now, applying the following similarity transformation variables

14 $$\begin{aligned} \psi & = \sqrt {2\vartheta lU_{w} } e^{x/2l} f\left( {\eta ,\tau } \right); \eta = y\sqrt {\frac{{U_{w} }}{2\vartheta l}} e^{x/2l} ; \tau = \frac{{U_{w} }}{2l}e^{x/l} .t; \theta \left( {\eta ,\tau } \right) \\ & = \left( {T - T_{\infty } } \right)/\left( {T_{w} - T_{\infty } } \right). \\ \end{aligned}$$

Using (14) on Eqs. (12–13) produces

15 $$\begin{aligned} & \frac{{\partial^{3} f\left( {\eta , \tau } \right)}}{{\partial \eta^{3} }} + \xi_{1} \xi_{2} \left\{ {\frac{{\partial^{2} f\left( {\eta , \tau } \right)}}{{\partial \eta^{2} }}f\left( {\eta , \tau } \right) - 2\left( {\frac{{\partial f\left( {\eta , \tau } \right)}}{\partial \eta }} \right)^{2} - \frac{{\partial^{2} f\left( {\eta , \tau } \right)}}{\partial \tau \partial \eta } + 2\lambda_{1} \left( {\beta_{hnf} /\beta_{f} } \right)\theta \left( {\eta , \tau } \right)} \right\} \\ & \quad - \frac{{\sigma_{hnf} }}{{\sigma_{f} }}\xi_{2} \frac{{\partial f\left( {\eta , \tau } \right)}}{\partial \eta } - K\frac{{\partial f\left( {\eta , \tau } \right)}}{\partial \eta } = 0 \\ \end{aligned}$$

16 $$\frac{1}{{Pr\xi_{3} }}\left[ {\left( {k_{hnf} /k_{f} } \right) + \frac{4Rd}{3}} \right] \frac{{\partial^{2} \theta \left( {\eta ,\tau } \right)}}{{\partial \eta^{2} }} + f\left( {\eta , \tau } \right)\frac{{\partial \theta \left( {\eta ,\tau } \right)}}{\partial \eta } - 4\frac{{\partial f\left( {\eta , \tau } \right)}}{\partial \eta }\theta \left( {\eta ,\tau } \right) - \frac{{\partial \theta \left( {\eta ,\tau } \right)}}{\partial \tau } = 0$$

with corresponding boundary conditions

17 $$\left\{ {\begin{array}{*{20}l} {f\left( {0,\tau } \right) = S,\frac{\partial f}{{\partial \eta }}\left( {0,\tau } \right) = - 1 + \delta \frac{{\partial^{2} f\left( {0, \tau } \right)}}{{\partial \eta^{2} }},\theta \left( {0,\tau } \right) = 1 + \delta_{T} \frac{{\partial \theta \left( {0,\tau } \right)}}{\partial \eta }} \hfill \\ {f^{\prime } \left( {\eta ,\tau } \right) \to 0, \theta \left( {\eta ,\tau } \right) \to 0\;{\text{as}}\;\eta \to \infty } \hfill \\ \end{array} } \right..$$

To test stability analysis of solutions, some small perturbation functions are assumed $f\left( \eta \right) = f_{0} \left( \eta \right)\;{\text{and}}\;\theta \left( \eta \right) = \theta_{0} \left( \eta \right)$ such as

18 $$\theta \left( {\eta ,\tau } \right) = \theta_{0} \left( \eta \right) + e^{ - \varepsilon \tau } G\left( {\eta ,\tau } \right);f\left( {\eta ,\tau } \right) = f_{0} \left( \eta \right) + e^{ - \varepsilon \tau } F\left( {\eta ,\tau } \right)$$

here $G\left( {\eta ,\tau } \right)$ and $F\left( {\eta ,\tau } \right)$ and are the small concerned to $\theta_{0} \left( \eta \right)$. and $f_{0} \left( \eta \right)$ and $\varepsilon^{\prime \prime }$ is the unknown eigenvalue. Putting Eq. (18) into Eqs. (15–17) where solutions $f\left( \eta \right) = f_{0} \left( \eta \right)$ and $\theta \left( \eta \right) = \theta_{0} \left( \eta \right)$ of steady state Eqs. (8–9) are found by setting τ = 0. Thus, we get

19 $$F_{0}^{\prime \prime \prime } + \xi_{1} \left\{ {f_{0} F_{0}^{\prime \prime } + F_{0} f_{0}^{\prime \prime } - 4f_{0}^{\prime } F_{0}^{\prime } + 2\lambda_{1} \left( {\beta_{hnf} /\beta_{f} } \right)G_{0} + \varepsilon F_{0}^{\prime } } \right\} - \frac{{\sigma_{hnf} }}{{\sigma_{f} }}\xi_{2} F_{0}^{\prime } - KF_{0}^{\prime } = 0$$

20 $$\frac{1}{{Pr\xi_{3} }}\left[ {\left( {k_{hnf} /k_{f} } \right) + \frac{4Rd}{3}} \right]G_{0}^{\prime \prime } + f_{0} G_{0}^{\prime } + F_{0} \theta_{0}^{\prime } - 4f_{0}^{\prime } G_{0} - 4F_{0}^{\prime } \theta_{0} + \frac{2Ec}{{\xi_{2} \xi_{3} }}f_{0}^{\prime \prime } F_{0}^{\prime \prime } + \varepsilon G_{0} = 0$$

whose boundary conditions are

21 $$\left\{ {\begin{array}{*{20}l} {F_{0} \left( 0 \right) = 0, F_{0}^{\prime } \left( 0 \right) = \delta F_{0}^{\prime \prime } \left( 0 \right), G_{0} \left( 0 \right) = \delta_{T} G_{0}^{\prime } \left( 0 \right)} \hfill \\ {F_{0}^{\prime } \left( \eta \right) \to 0, G_{0} \left( \eta \right) \to 0\;{\text{as}}\;\eta \to \infty } \hfill \\ \end{array} } \right..$$

The boundary condition should be reduced to the initial condition to find the $\varepsilon_{1}$. Hence, we reduced $F_{0}^{\prime } \left( \eta \right) \to 0$ as η → ∞ into $F_{0}^{\prime \prime } \left( 0 \right) = 1$.

## Validation of results

Before starting to discuss the results of the current study, we have compared coding of a numerical method to ensure that our computer code works properly. The results are verified in Table 3 for limiting flow constraints with work of Lund et al.^21^. A good agreement has been noticed of current results with investigation of Lund et al.^21^. The results are further verified in Table 4 with outcomes of Yan et al.^22^. An outstanding measurement between both results are reported.

**Table 3:** Values of $f^{\prime \prime } \left( 0 \right)$ and $\theta^{\prime } \left( 0 \right)$ for the various values of $Pr$ by keeping $S = 5, \phi_{{{\text{Al}}_{2} {\text{O}}_{3} }} = \phi_{{{\text{Cu}}}} = 0, \lambda_{1} = - 0.5, Rd = K = M = \delta = \delta_{T} = 0.$

$Pr$	Lund et al.^21^		Present	Results
	$f^{\prime \prime } \left( 0 \right)$	$\theta^{\prime } \left( 0 \right)$	$f^{\prime \prime } \left( 0 \right)$	$\theta^{\prime } \left( 0 \right)$
1	4.449203	− 4.447507	4.449203	− 4.447507
1.6	4.540536	− 7.334577	4.540536	− 7.334577
2	4.570372	− 9.284828	4.570372	− 9.284828
2.4	4.590011	− 11.247347	4.590011	− 11.247347
6.2			4.648148	− 30.10742

**Table 4:** Values of $f^{\prime \prime } \left( 0 \right)$ for the various values of $\phi_{{{\text{Cu}}}}$ by keeping $\phi_{{{\text{Al}}_{2} {\text{O}}_{3} }} = 0.1,\lambda_{1} = \delta = K = M = 0$ and $S = 3$.

$\phi_{{{\text{Cu}}}}$	Yan et al.^22^		Present	Results
	Upper branch	Lower branch	Upper branch	Lower branch
	$f^{\prime \prime } \left( 0 \right)$		$f^{\prime \prime } \left( 0 \right)$	
0.01	2.48626	− 1.10767	2.48626	− 1.10767
0.05	2.81888	− 1.62610	2.81888	− 1.62610
0.1	3.07486	− 2.08072	3.07486	− 2.08072

## Results and discussion

**Figure 2:**
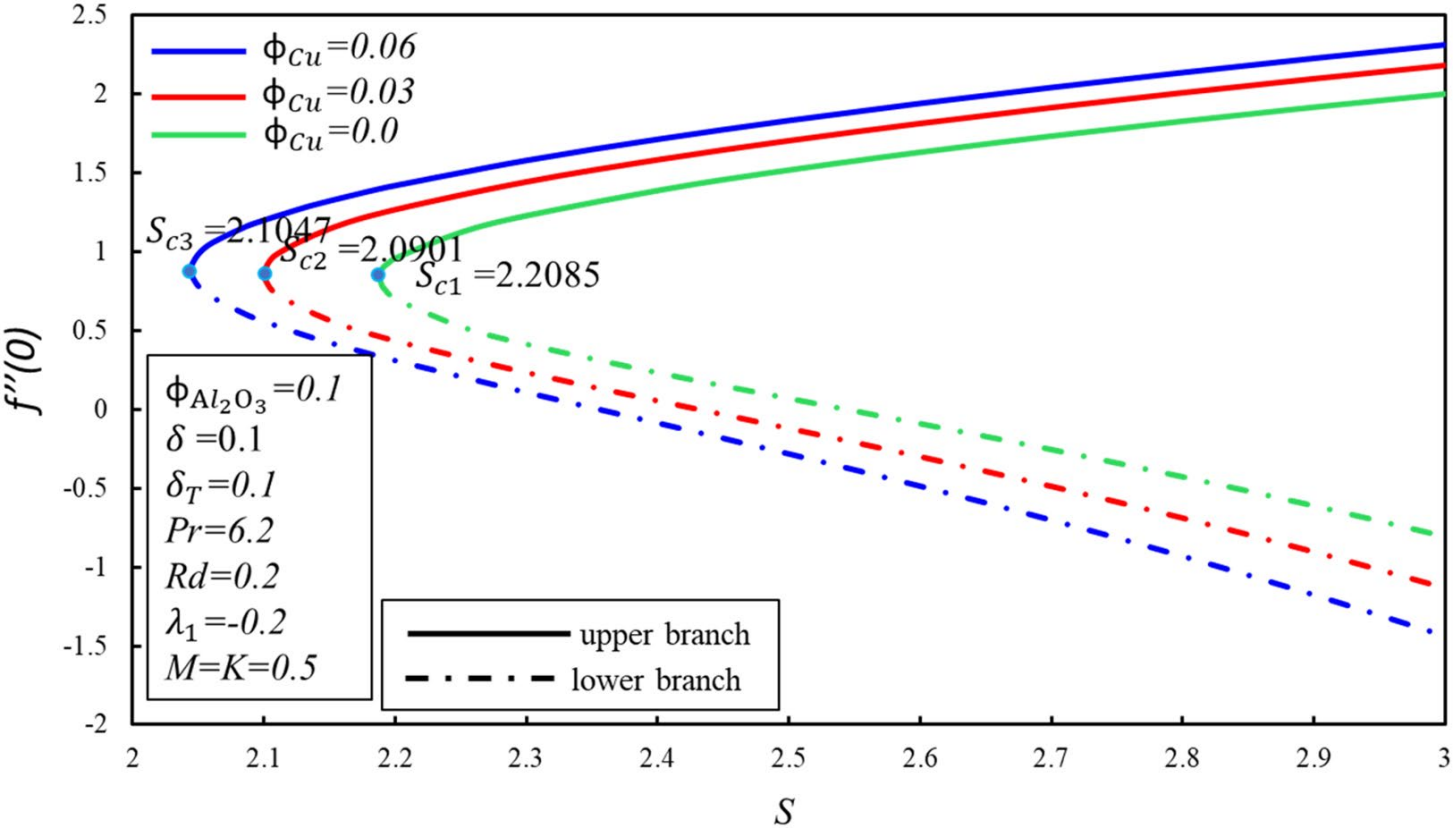
Behavior of $f^{\prime \prime } \left( 0 \right)$ in impact of $\phi_{{{\text{Cu}}}}$.

**Figure 3:**
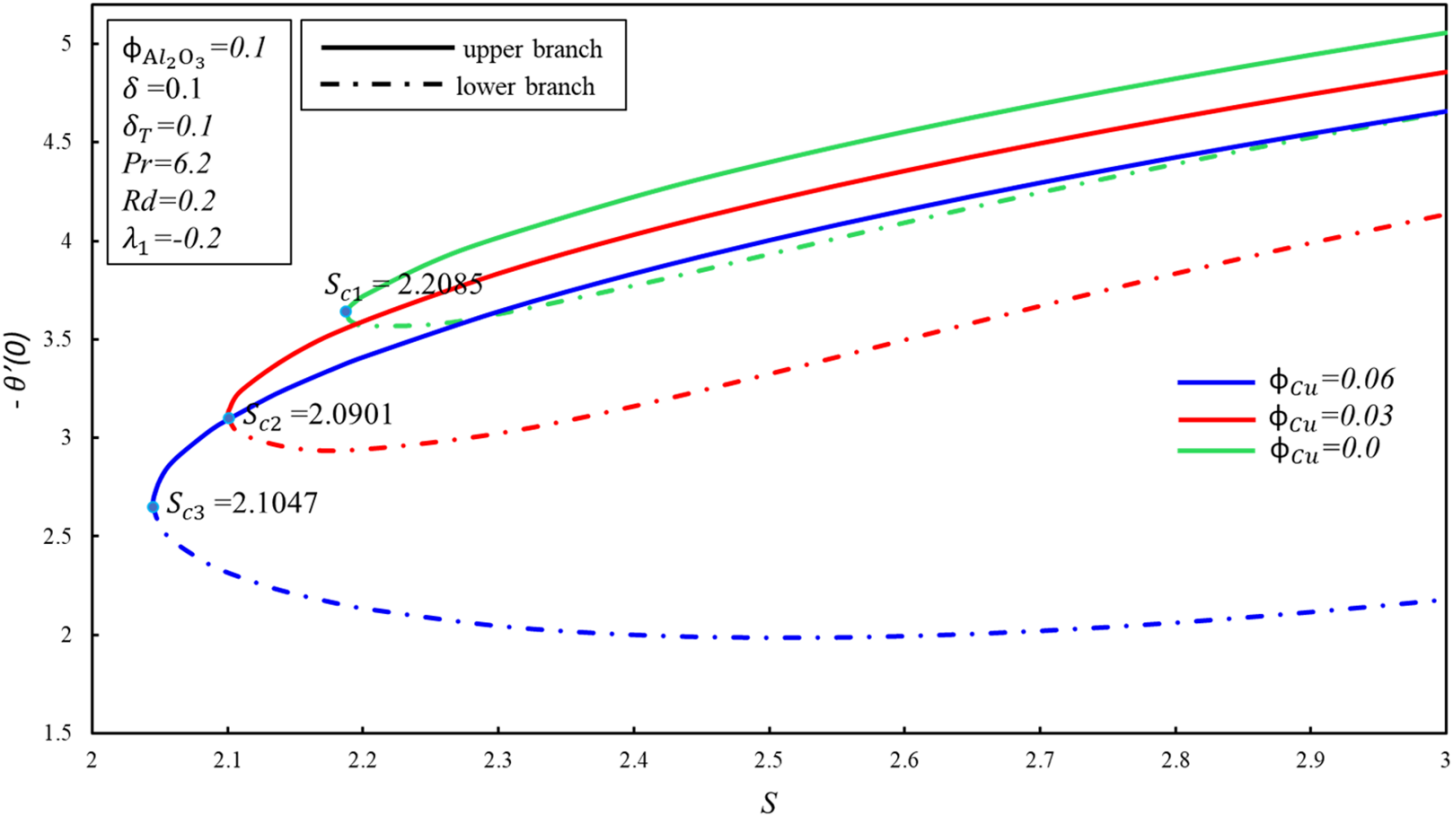
Behavior of $- \theta^{\prime } \left( 0 \right)$ in impact of $\phi_{{{\text{Cu}}}}$.

The system of nonlinear ODEs (6–7) subject to boundary condition (9) has been successfully solved using shooting method with 4th order of Runge Kutta process in Maple software. This method has been widely used by numerous academics and scholars to solve fluid flow problems. All over the figures, the solutions duality has been gotten by using various initial guessing for $f^{\prime \prime } \left( 0 \right)$ and $\theta^{\prime } \left( 0 \right)$ in which all the profiles of velocity and temperature satisfied the boundary condition $\eta \to \infty$ asymptotically. Throughout this study, we kept a Prandtl number $Pr = 6.2$ for water at 25 °C and the $\phi_{{{\text{Al}}_{2} {\text{O}}_{3} }} = 0.1$ as proposed by Devi and Devi^24^. The range of $\phi_{{{\text{Cu}}}}$ is 0 to 0.06. Figures 2 and 3 show the effect of $\phi_{{{\text{Cu}}}}$ on behavior of skin friction coefficient $f^{\prime \prime } \left( 0 \right)$ and rate of heat transfer $- \theta^{\prime } \left( 0 \right)$, respectively. Both figures portray the dropping characters as $\phi_{Cu}$ rises in the lower branch. In upper branch, $f^{\prime \prime } \left( 0 \right)$ increases with $\phi_{Cu}$ and $S$. On the other hand, $- \theta^{\prime } \left( 0 \right)$ rises in both branches when $S$ enhances. It is observed that the fluid is flowing towards $S$ till it arrives a point $S_{ci}$ where $i = 1,2,3$, $S_{ci}$ is the critical point of $S$ where the connection of the upper and lower branch exists. No branch exists when $S < S_{ci}$. It is worth mentioning that when $\phi_{{{\text{Cu}}}} = 0$, it is purely ${\text{Al}}_{2} {\text{O}}_{3}$ water based nanofluid and $S_{c1} = 2.2085$, after that 3% of $\phi_{Cu}$ is added and got $S_{c2} = 2.0901$. Additionally, the value of $S_{c3}$ seems to increase as an addition of 6% of the solid volume fraction of $\phi_{{{\text{Cu}}}}$ in the hybrid nanofluid. Furthermore, increasing in $\phi_{{{\text{Cu}}}}$ extended the separation of layer and the branch range seems to release.

**Figure 4:**
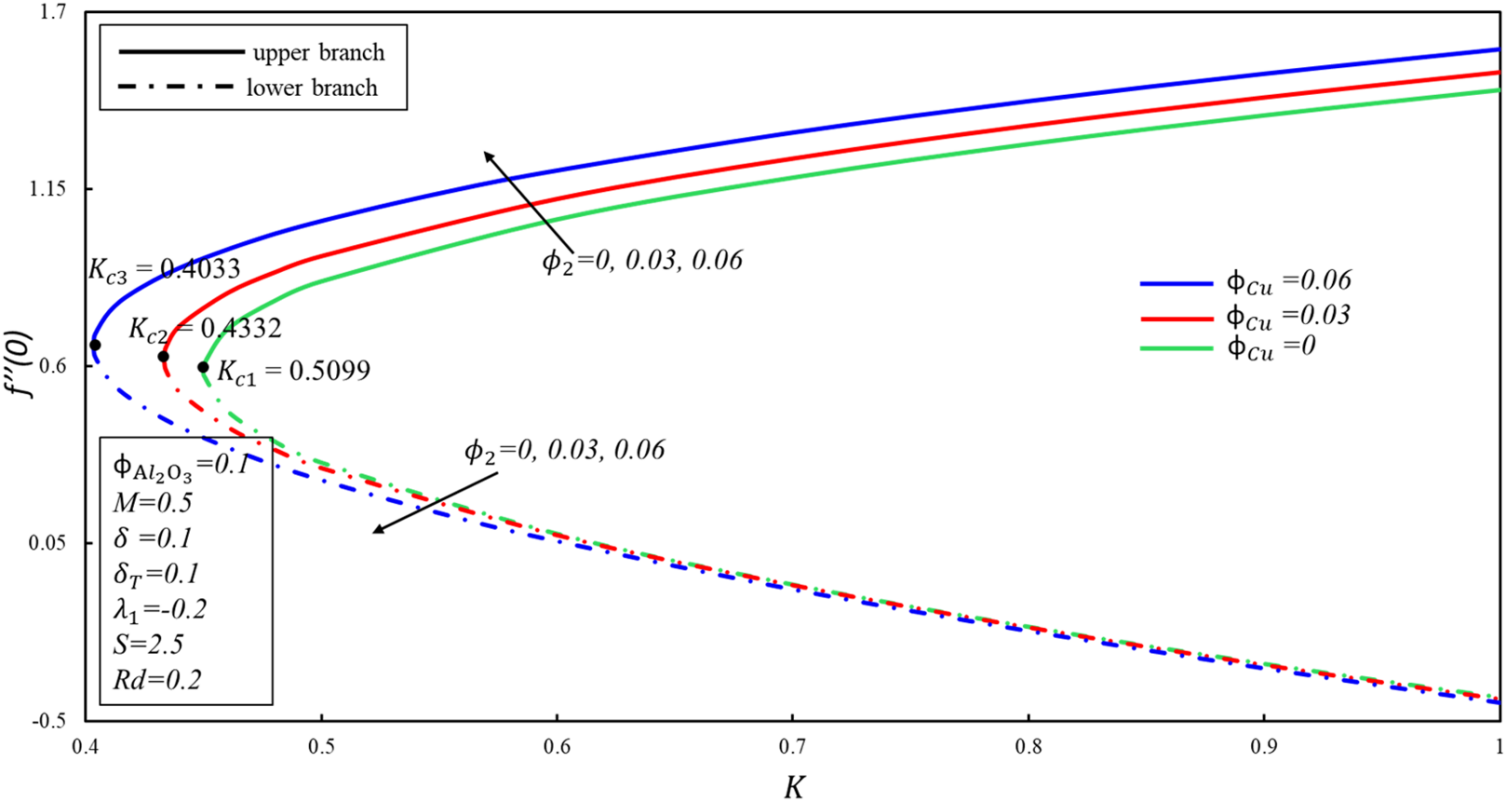
Behavior of $f^{\prime \prime } \left( 0 \right)$ in impact of $\phi_{{{\text{Cu}}}}$.

**Figure 5:**
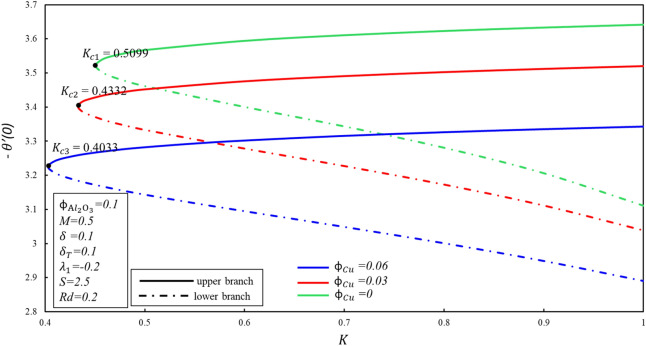
Behavior of $- \theta^{\prime } \left( 0 \right)$ in impact of $\phi_{{{\text{Cu}}}}$.

**Figure 6:**
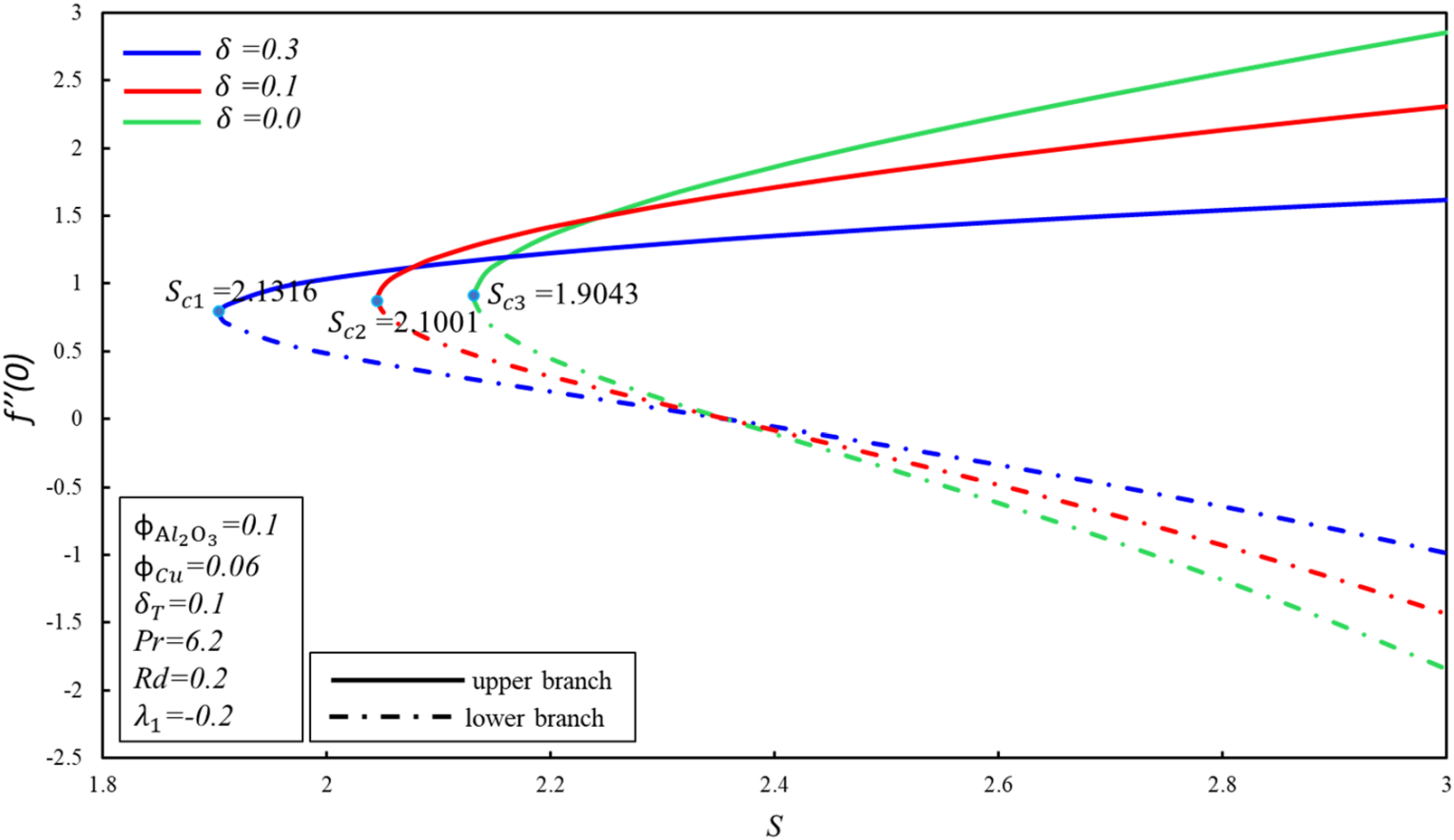
Behavior of $f^{\prime \prime } \left( 0 \right)$ in impact of $\delta$.

**Figure 7:**
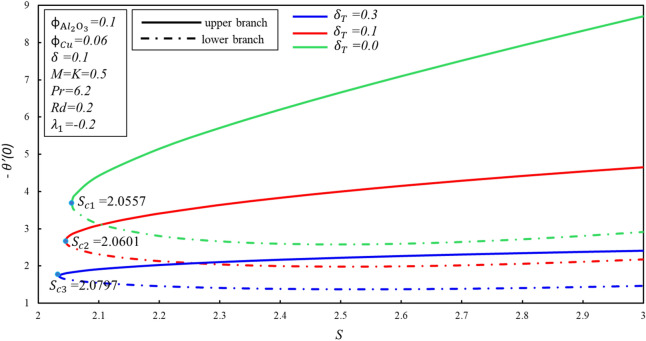
Behavior of $- \theta^{\prime } \left( 0 \right)$ in impact of $\delta_{T}$.

Figures 4 and 5 depicted the porosity parameter $K$ on the magnitude of $f^{\prime \prime } \left( 0 \right)$ and $- \theta^{\prime } \left( 0 \right)$ for different values of $\phi_{{{\text{Cu}}}}$. The corresponding critical values of $\phi_{{{\text{Cu}}}}$ are $K_{c}$ and $K_{c}$ denotes critical point at which both branches exist. Dual branches are noted as $K_{C} \le K$ and no branch occurs when $K_{C} > K$. It is indicating that ahead $K_{C}$ values, no branch exists. Reduced skin friction ($f^{\prime \prime } \left( 0 \right)$) raises when $\phi_{{{\text{Cu}}}}$ and $K$ raises in stable branch, although it decreases in second branch when the-two applied parameters raise. A decreasing heat transfer ($- \theta^{\prime } \left( 0 \right)$) decrease in the-two branches when $\phi_{{{\text{Cu}}}}$ enhances, though opposite movement is noticed while $K$ enhances in the upper branch by keeping the constant values $\phi_{{{\text{Cu}}}}$. It is observed that the porosity values are substantial to evaluate the existence of non-unique solutions. The various sort of velocity slip condition on $f^{\prime \prime } \left( 0 \right)$ is highlighted in Fig. 6. Partial kinds of slip conditions are examined in this study where $\delta$ shows the effect of the velocity slip condition of the first order and $\delta_{T}$ indicates the thermal slip effect of the first order. It is evidently pragmatic that no-slip condition (when $\delta = 0$) has a lower effect on the boundary layer separation of the hybrid nanofluid as compared to the velocity slip. The flow of hybrid fluid is flowed till a critical point $S_{ci}$, while no flow of fluid is possible when $S < S_{ci}$. When suction velocity slip increase, skin friction increases (decreases) in upper branch. The lower branch of $f^{\prime \prime } \left( 0 \right)$ exhibits the inverse trend. Figure 7 demonstrates the effect of thermal $\left( {\delta_{T} } \right)$ slip with $S$ on $- \theta^{\prime } \left( 0 \right)$. Suction is often used to increase performance of diffusers with strong compression ratios of the flow. The thermal condition value is improved by developing the early layer separation. As it may be clearly seen, the magnitude of the thermal slip decreases because of suction before the critical value $S_{ci}$ is raised. When $\delta_{T} = 0, 0.1, 0.3$, we have $S_{ci} = 2.0557, 2.0601, 2.0797$ respectively. It is noted that the branch duality occurs when $S < S_{ci}$ and no branch exists beyond $S_{ci}$. Heat transfer reduces in both branches when $\delta_{T}$ is improved. Substantially, this decreasing trend is due to the fact that heat is transferring fast from the surface to cold areas of the hybrid nanofluid.

**Figure 8:**
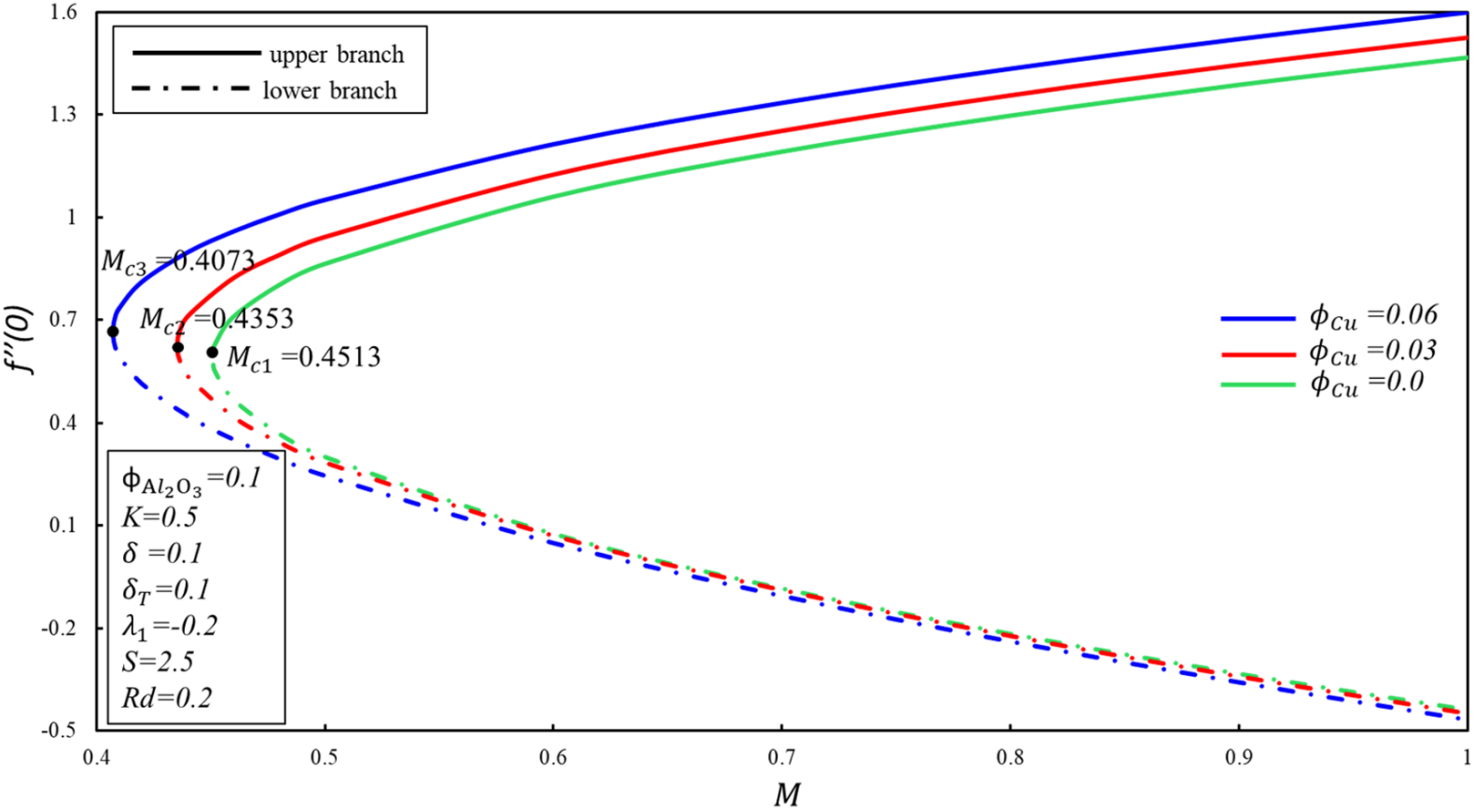
Behavior of $f^{\prime \prime } \left( 0 \right)$ in impact of $\phi_{{{\text{Cu}}}}$.

**Figure 9:**
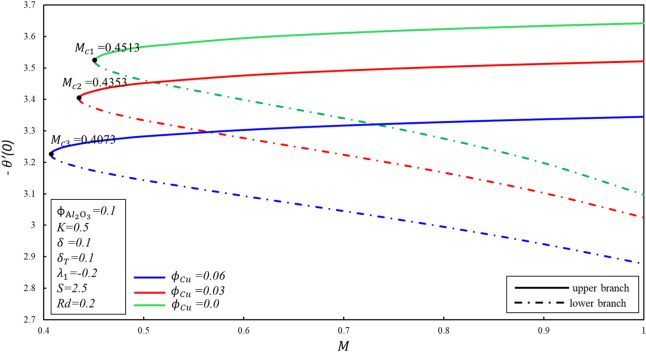
Behavior of $- \theta^{\prime } \left( 0 \right)$ in impact of $\phi_{{{\text{Cu}}}}$.

Figures 8 and 9 display the variations of $f^{\prime \prime } \left( 0 \right)$ and $- \theta^{\prime } \left( 0 \right)$ at different values of $M$. $M_{c} = 0.4513,0.4353, 0.4073$ corresponds to the critical value of the parameter $\phi_{{{\text{Cu}}}} = 0, 0.03, 0.06$, here $M_{c}$ is the juncture where both branches meet. In the case of $M < M_{c}$, no branch occurs, and dual branches are marked as $M \ge M_{C}$. The boundary layer estimation is no longer reliable when the critical value is exceeded. Reduced skin friction ($f^{\prime \prime } \left( 0 \right)$) enhances when $\emptyset_{2}$ is enhanced in upper branch, whereas it reduces in lower branch. In addition, $f^{\prime \prime } \left( 0 \right)$ enhances in upper branch while $M$ enhances by keeping fixed value of $\phi_{{{\text{Cu}}}}$, although reverse movement is observed in lower branch. This fact is associated to cause that Lorenz force repressed the vortex developed by shrunk surface within boundary layer. Reduced heat transfer ($- \theta^{\prime } \left( 0 \right)$) enhances for upper branch when magnitude of $M$ raises, although reverse movement is identified for lower branch. It should be noted that when $\phi_{{{\text{Cu}}}} = 0$, the equations accept the alumina nanofluid model.

**Figure 10:**
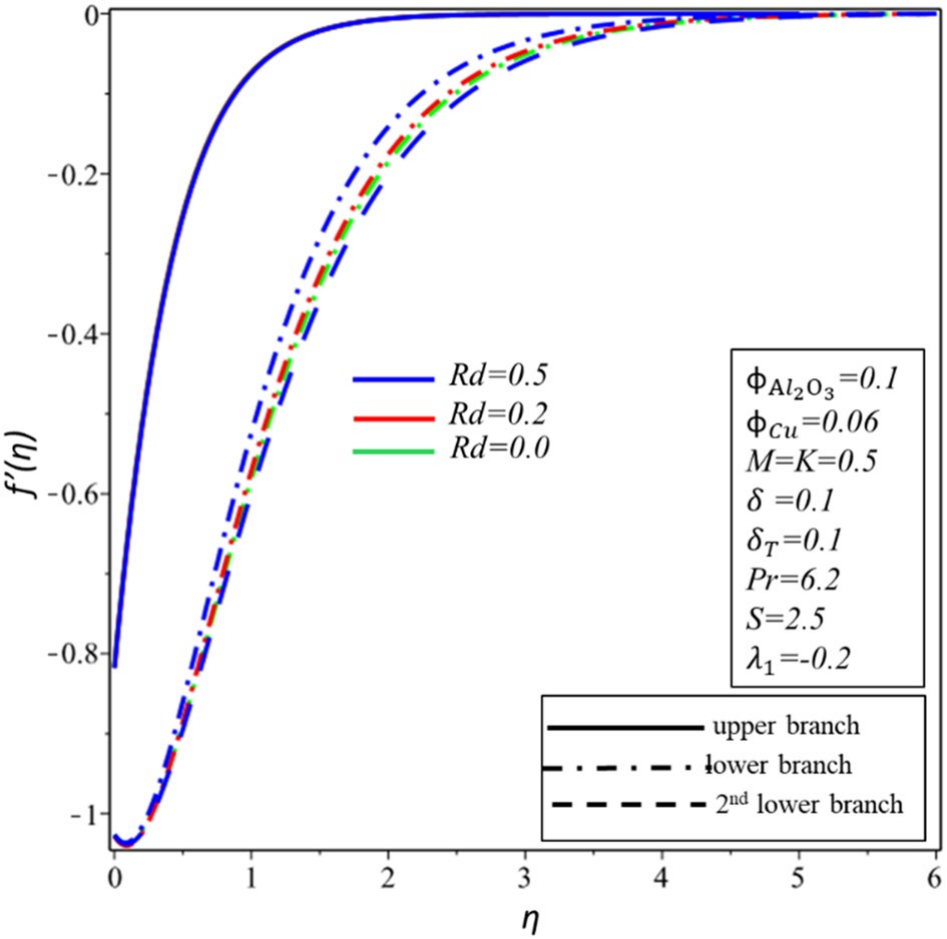
Behavior of $f^{\prime } \left( \eta \right)$ in impact of $Rd$.

**Figure 11:**
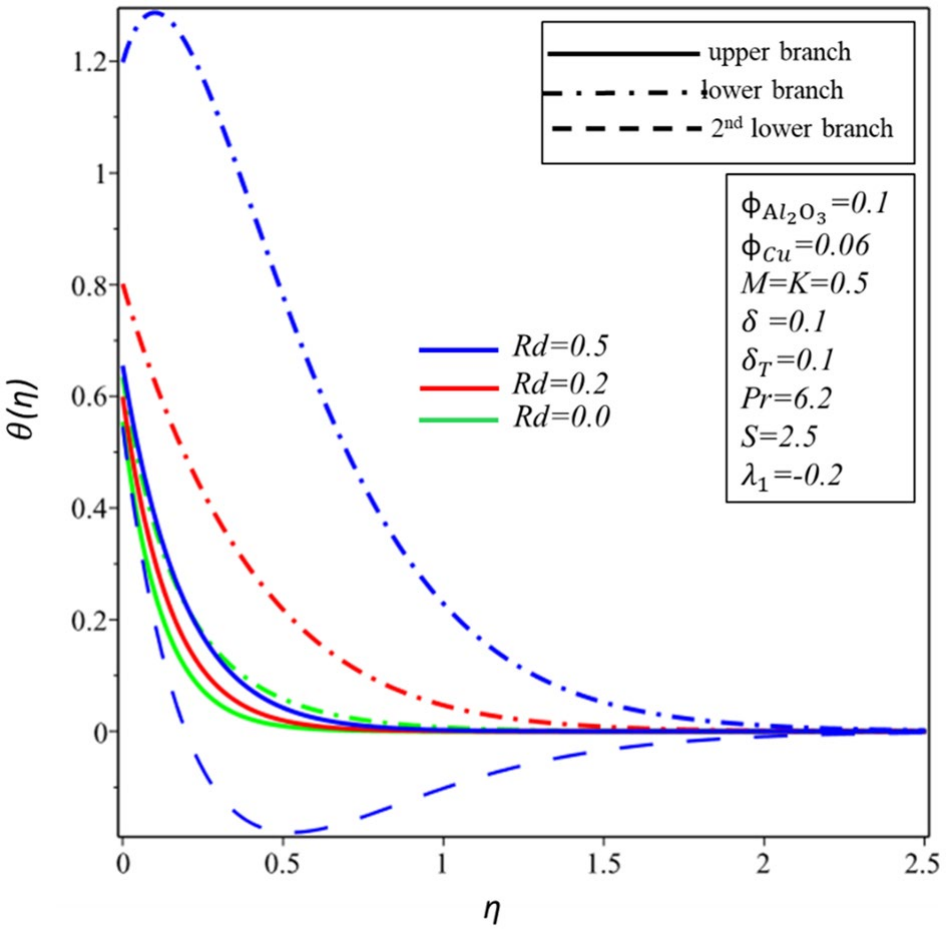
Behavior of $\theta \left( \eta \right)$ in impact of $Rd$.

**Figure 12:**
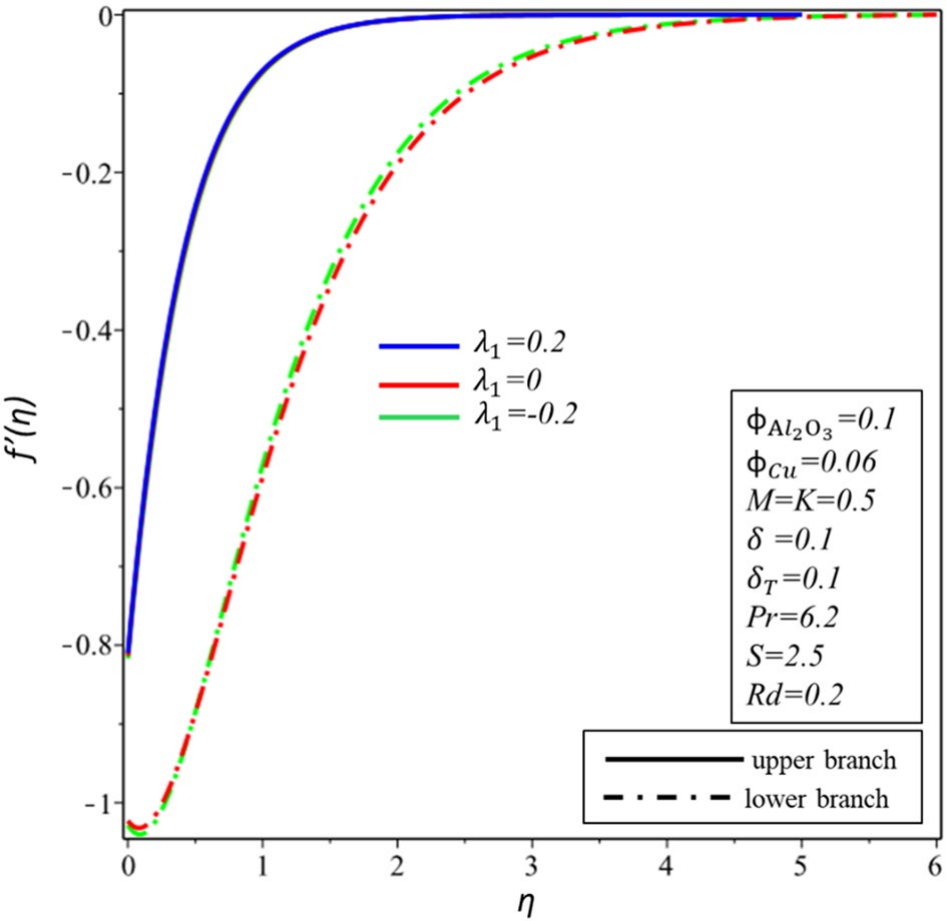
Behavior of $f^{\prime } \left( \eta \right)$ in impact of $\lambda_{1}$.

**Figure 13:**
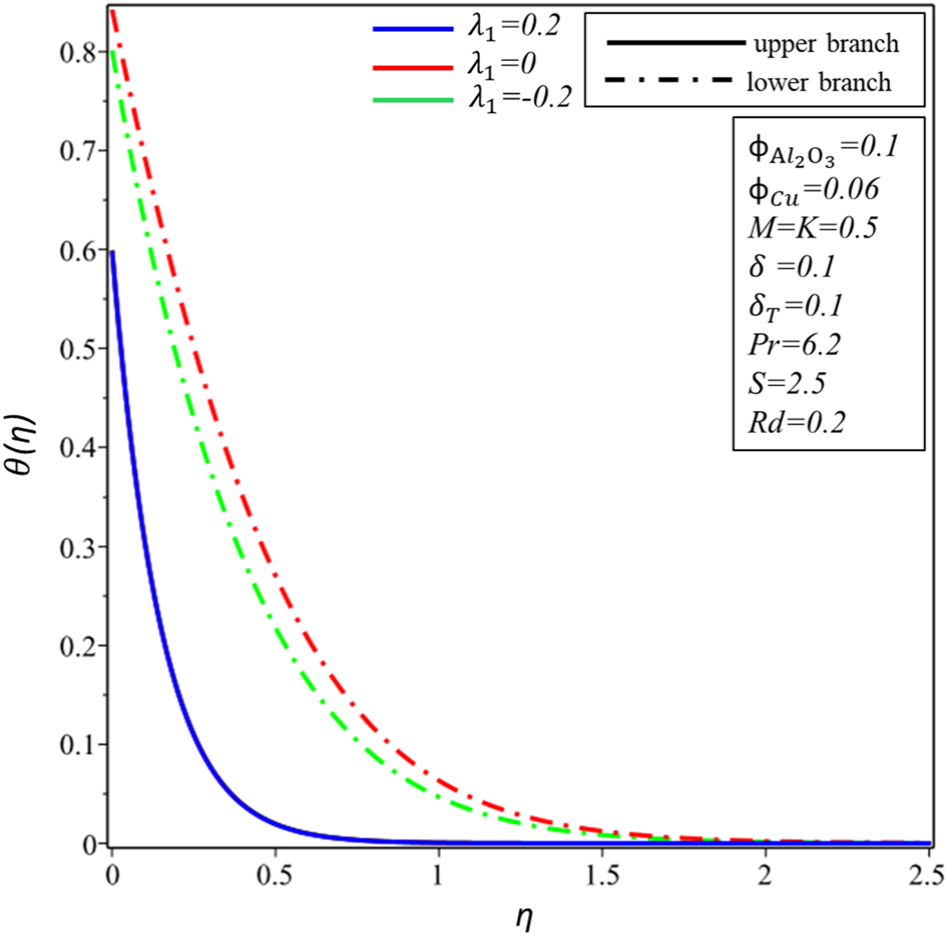
Behavior of $\theta \left( \eta \right)$ in impact of $\lambda_{1}$.

**Figure 14:**
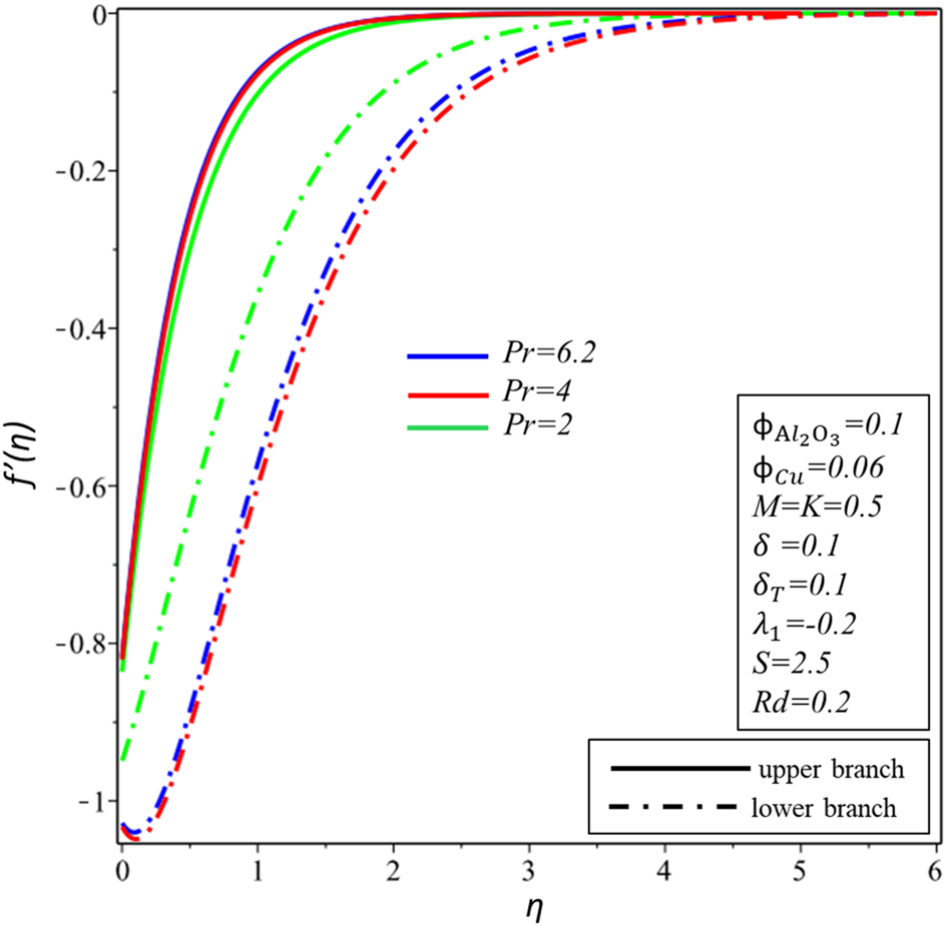
Behavior of $f^{\prime } \left( \eta \right)$ in impact of $Pr$.

**Figure 15:**
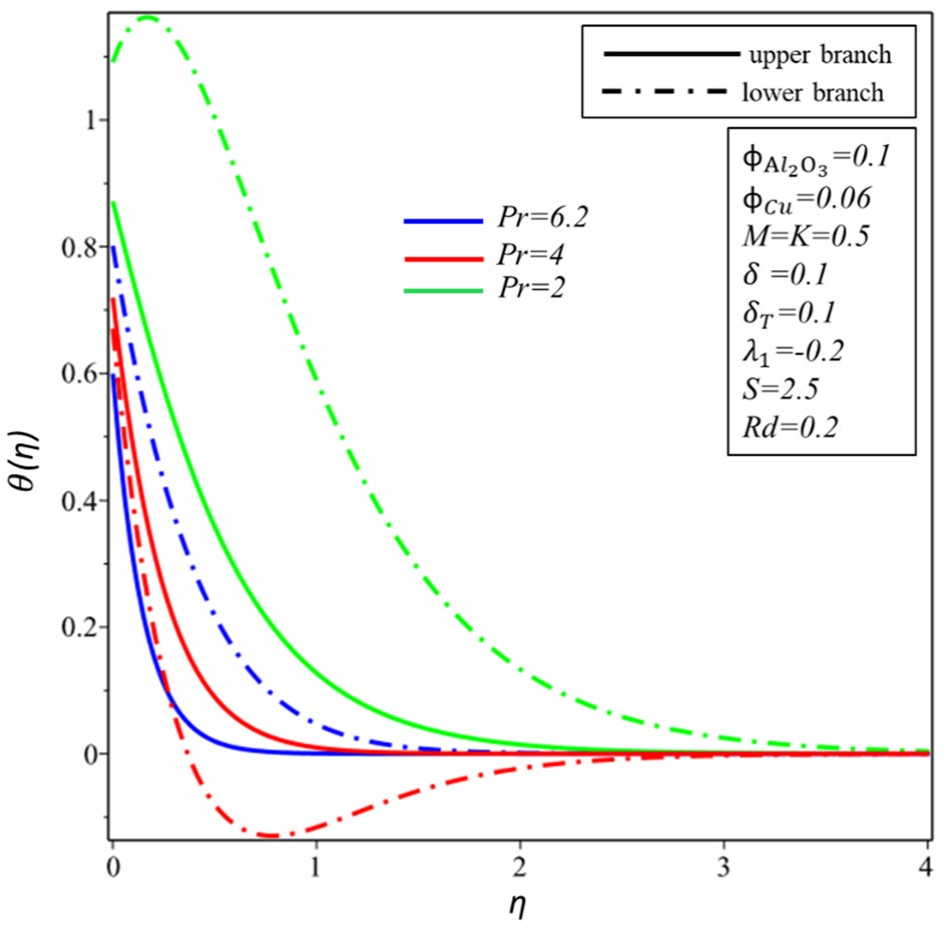
Behavior of $\theta \left( \eta \right)$ in impact of $Pr$.

Figures 10 and 11 depict the effect of $Rd$ on the velocity distribution $f^{\prime } \left( \eta \right)$ and temperature distribution $\theta \left( \eta \right)$. These figures indicate that the boundary conditions for upper and lower branches are asymptotic and thus endorse the graphic findings stated in Figs. 2, 3, 4, 5, 6, 7, 8 and 9. It is worth mentioning here that triple branches exist in limited values of the applied parameters. See Fig. 10, three branches exist when $Rd = 0.5$. The velocity distribution for the lower branch decreases expressively while no change is observed in the upper branch. Additionally, it is noted that temperature distribution constantly rises in the upper and lower branches with a rising value of $Rd$. The plots of $f^{\prime } \left( \eta \right)$ and $\theta \left( \eta \right)$ against $\lambda_{1}$ are shown in Figs. 12 and 13, respectively. It is revealed that the upper branch of $f^{\prime } \left( \eta \right)$ and $\theta \left( \eta \right)$ neither increase nor decrease with the increasing values of $\lambda_{1}$, but $f^{\prime } \left( \eta \right)$ and $\theta \left( \eta \right)$ are increasing functions of $\lambda_{1}$ for the lower branch. Besides, these graphs display that branches duality are conceivable for buoyancy assisting flow. On the other hand, a single branch exists for the opposing flow case. It is worth to define them here, assisting (opposing) flow happens when the force of buoyancy and the velocity of the surface are in the similar (opposite) direction. From Figs. 14 and 15, the decreasing behavior of upper branch of $f^{\prime } \left( \eta \right)$ and $\theta \left( \eta \right)$ is observed. For $f^{\prime } \left( \eta \right)$ ($\theta \left( \eta \right)$), lower branch increases (decreases) in the range of $2 \le Pr \le 4$ and decreases (increases) in the range of $4 < Pr \le 6.2$. Table 5 is constructed to display the values of the smallest eigenvalue $\varepsilon_{1} .$ It is obtained from the table that upper branch is the stable one.

**Table 5:** The numerical values of $\varepsilon_{1}$ for various values of suction where $\phi_{{{\text{Al}}_{2} {\text{O}}_{3} }} = 0.1, \phi_{{{\text{Cu}}}} = 0.06, \lambda_{1} = - 0.2, Rd = 0.2, \delta = \delta_{T} = 0.1$.

*S*	$\varepsilon_{1}$	
	Upper branch	Lower branch
3	2.17111	− 1.9403
2.8	1.97369	− 1.7038
2.6	1.59794	− 1.4271
2.4	1.05140	− 0.9274
2.2	0.98623	− 0.86513
2.0447	0.00538	− 0.0948

## Conclusion

The radiative flow of hybrid nanofluid influences by multiple slip constraints has been addressed for vertical plate. The thermal results are observed with applications of mixed convection, magnetic force and porous media space. The thermal stability of model is checked and ensured. The validation of findings was carried out for limited situations where the current numerical outcomes have been well correlated with the previously published results.

- The hybrid nanoparticles addition comprehensively enhanced the characteristics of water base fluid.
- Dual even triple branches have been shown to be feasible with a confident range of applied parameters.
- The rate of heat transfer has decelerated with rising values of thermal slip condition. In addition, it has been revealed that the branches bifurcation has existed when $\lambda_{1} = - 0.2$.
- The flow of hybrid fluid is flowed till a critical point $S_{ci}$, while no flow of fluid is possible when $S < S_{ci}$.
- The study of temporal stability has revealed that only one of the two branches is reliable and stable, whilst the other is unreliable in the long term.

## Data Availability

The datasets used and/or analysed during the current study available from the corresponding author on reasonable request.

## List of symbols

$K^{*}$: Permeability of porous medium
$T$: Temperature of fluid
$T_{\infty }$: Free stream temperature
$$\rho_{hnf}$$: Density
$$k_{hnf}$$: Thermal conductivity
$$u_{w}$$: Surface velocity
$D$: Thermal slip factor
$$\lambda_{1}$$: I mixed parameter
$K$: Parameter of porosity
$Nu_{x}$: Local Nusselt number
$\delta_{T}$: Thermal slip parameter
$Rd$: Thermal radiation parameter
$B_{0}$: Magnetic field c strength
$T_{w} \left( x \right)$: Temperature of surface
$\left( {\rho c_{p} } \right)_{hnf}$: Effective heat capacity
$$\sigma_{hnf}$$: Electrical conductivity
$$\mu_{hnf}$$: Viscosity
$A$: Velocity slip factor
$S$: Parameter of blowing/suction
$$M$$: Hartmann number
$\delta$: Velocity slip parameter
$$Pr$$: Prandtl number
$C_{f}$: Skin friction coefficient
